# Type 2 diabetes and HbA_1c_ are independently associated with wider retinal arterioles: the Maastricht study

**DOI:** 10.1007/s00125-020-05146-z

**Published:** 2020-05-08

**Authors:** Wenjie Li, Miranda T. Schram, Tos T. J. M. Berendschot, Carroll A. B. Webers, Abraham A. Kroon, Carla J. H. van der Kallen, Ronald M. A. Henry, Nicolaas C. Schaper, Fan Huang, Behdad Dashtbozorg, Tao Tan, Jiong Zhang, Samaneh Abbasi-Sureshjani, Bart M. ter Haar Romeny, Coen D. A. Stehouwer, Alfons J. H. M. Houben

**Affiliations:** 1 CARIM School for Cardiovascular Diseases, Maastricht University, Maastricht, the Netherlands; 2 Department of Internal Medicine, Maastricht University Medical Center+, P. Debyelaan 25, 6229HX Maastricht, the Netherlands; 3 University Eye Clinic Maastricht, Maastricht University Medical Center+, Maastricht, the Netherlands; 4 CAPHRI Care and Public Health Research Institute, Maastricht University, Maastricht, the Netherlands; 5 Department of Biomedical Engineering, Eindhoven University of Technology, Eindhoven, the Netherlands; 6 USC Stevens Neuroimaging and Informatics Institute, Keck School of Medicine of USC, University of Southern California, Los Angeles, CA USA

**Keywords:** Clinical diabetes, Epidemiology, Human, Microvascular disease, Pathogenic mechanism, Pathophysiology/metabolism

## Abstract

**Aims/hypothesis:**

Retinal microvascular diameters are biomarkers of cardio-metabolic risk. However, the association of (pre)diabetes with retinal microvascular diameters remains unclear. We aimed to investigate the association of prediabetes (impaired fasting glucose or impaired glucose tolerance) and type 2 diabetes with retinal microvascular diameters in a predominantly white population.

**Methods:**

In a population-based cohort study with oversampling of type 2 diabetes (*N* = 2876; *n* = 1630 normal glucose metabolism [NGM], *n* = 433 prediabetes and *n* = 813 type 2 diabetes, 51.2% men, aged 59.8 ± 8.2 years; 98.6% white), we determined retinal microvascular diameters (measurement unit as measured by retinal health information and notification system [RHINO] software) and glucose metabolism status (using OGTT). Associations were assessed with multivariable regression analyses adjusted for age, sex, waist circumference, smoking, systolic blood pressure, lipid profile and the use of lipid-modifying and/or antihypertensive medication.

**Results:**

Multivariable regression analyses showed a significant association for type 2 diabetes but not for prediabetes with arteriolar width (vs NGM; prediabetes: β = 0.62 [95%CI −1.58, 2.83]; type 2 diabetes: 2.89 [0.69, 5.08]; measurement unit); however, there was a linear trend for the arteriolar width across glucose metabolism status (*p* for trend = 0.013). The association with wider venules was not statistically significant (prediabetes: 2.40 [−1.03, 5.84]; type 2 diabetes: 2.87 [−0.55, 6.29], *p* for trend = 0.083; measurement unit). Higher HbA_1c_ levels were associated with wider retinal arterioles (standardised β = 0.043 [95% CI 0.00002, 0.085]; *p* = 0.050) but the association with wider venules did not reach statistical significance (0.037 [−0.006, 0.080]; *p* = 0.092) after adjustment for potential confounders.

**Conclusions/interpretation:**

Type 2 diabetes, higher levels of HbA_1c_ and, possibly, prediabetes, are independently associated with wider retinal arterioles in a predominantly white population. These findings indicate that microvascular dysfunction is an early phenomenon in impaired glucose metabolism.

**Electronic supplementary material:**

The online version of this article (10.1007/s00125-020-05146-z) contains peer-reviewed but unedited supplementary material, which is available to authorised users.

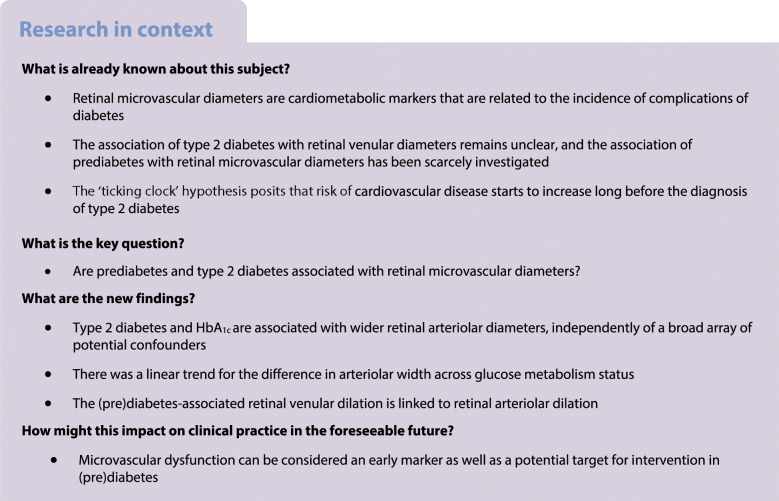



## Introduction

The worldwide epidemic of diabetes and its complications necessitates identification of early pathophysiological changes in the development of complications, as an essential requirement for risk assessment as well as for the design of interventions.

In recent studies, retinal microvascular diameters have been shown to be closely related to incidence of complications of diabetes, including retinopathy, nephropathy and stroke [1, 2], suggesting a role for early retinal changes in assessment of risk of these complications. However, the association of (pre)diabetes with retinal microvascular diameters remains unclear. Although type 2 diabetes has been associated with wider retinal arterioles in the majority of studies, an association of type 2 diabetes with wider retinal venules has only been found in non-white individuals [3–9]. Moreover, few studies have investigated the association of prediabetes with retinal microvascular diameters; those that have, also found that prediabetes was associated with wider retinal venules in non-white individuals only [3–6]. However, these studies [3–6] had significant limitations. For example, only one study used the gold standard of OGTT, rather than fasting glucose level or random glucose level, to define prediabetes and diabetes in a multi-ethnic population [5]. In addition, none adjusted for use of medication that is associated with glucose metabolism and microvascular function, such as antihypertensive drugs.

As it has been suggested that (pre)diabetes may be associated with both wider retinal arterioles and venules, the question arises whether, and, if so, how, these changes are related. Theoretically, wider arterioles could lead to wider venules via transmittance of greater microvascular pressure. Alternatively, or additionally, wider venules (if they are proven to be a marker for arteriole–venule shunting) could lead to wider arterioles through local tissue hypoxia. Recent studies on a multi-ethnic Asian population found that the association of (pre)diabetes with wider retinal arterioles was independent of retinal venular diameters, while the association with wider retinal venules was not independent of retinal arteriolar diameters [4, 7, 8], which supports the hypothesis that (pre)diabetes-associated retinal venular dilation is linked to retinal arteriolar dilation. However, these associations have not been studied in a white population.

Therefore, in this population-based cohort study, we investigated the associations of OGTT-based glucose metabolism status (normal glucose status, prediabetes, type 2 diabetes) and measures of blood glucose with retinal microvascular diameters in a predominantly white population, taking into account a broad array of potential confounders. In addition, we explored whether retinal arteriolar and venular diameters were mutually related.

## Methods

### Study population and design

We used data from The Maastricht Study, an observational, prospective population-based cohort study. The rationale and methodology have been described previously [10]. In brief, the study focuses on the aetiology, pathophysiology, complications and comorbidities of type 2 diabetes, and is characterised by an extensive phenotyping approach. All individuals aged between 40 and 75 years and living in the southern part of the Netherlands were eligible to participate. Participants were recruited through mass media campaigns and from the municipal registries and the regional Diabetes Patient Registry by postal mailing. Recruitment was stratified according to known type 2 diabetes status, with an oversampling of individuals with type 2 diabetes for reasons of efficiency. The present report includes cross-sectional data from the first 3451 participants, who completed the baseline survey between November 2010 and September 2013. The baseline examinations of each participant in the study were performed within a time window of 3 months (except for some participants in whom fundus photography was initially unavailable or in whom photos were of low quality; in these participants, fundus photography was obtained later; see below). The study was approved by the medical ethical committee of the Maastricht University Medical Center (NL31329.068.10) and the Minister of Health, Welfare and Sports of the Netherlands (permit 131088-105234-PG). All participants gave written informed consent. From the initial 3451 participants included, those with types of diabetes other than type 2 diabetes were excluded (*n* = 41). Of the remaining 3410 participants, retinal microvascular diameter data were available for 2924 participants, 48 of whom had data missing for one or more covariates. The main reasons for missing data were logistic (no equipment, no trained researcher available or technical failure), contraindications for the eye drops or fundus photographs of insufficient quality. The retinal microvascular diameter study population thus consisted of 2876 participants (ESM Fig. 1); fundus photography was obtained within the time window of 3 months in 2700 participants and after a mean of 47 months (range 34–57) after the date on which the retinal measurement was planned in 176 participants.

### Assessment of glucose metabolism status

To assess glucose metabolism status, all participants (except those who used insulin) underwent a standardised 2 h 75 g OGTT after an overnight fast. For safety reasons, participants with a fasting glucose level above 11.0 mmol/l, as determined by a finger prick test, did not undergo the OGTT. For these individuals, fasting glucose level and information about diabetes medication use were used to assess glucose metabolism status. Glucose metabolism status was defined according to the WHO 2006 criteria as normal glucose metabolism (NGM, fasting glucose <6.1 mmol/l; 2 h postload glucose <7.8 mmol/l), impaired fasting glucose and/or impaired glucose tolerance (combined as prediabetes, fasting glucose 6.1–7.0 mmol/l or 2 h postload glucose 7.8–11.1 mmol/l) and type 2 diabetes (fasting glucose ≥7.0 mmol/l or 2 h postload glucose ≥11.1 mmol/l) [11].

### Retinal photography and measurement of retinal microvascular diameters

All participants were asked to refrain from smoking and drinking caffeine-containing beverages for 3 h before the measurement. Participants were allowed to consume a light meal (breakfast or lunch) low in fat content at least 90 min before the start of the measurement [12]. For retinal measurements, fundus photography of both eyes was performed 15 min after the pupils had been dilated with tropicamide 0.5% and phenylephrine 2.5% (wt/vol.).

All fundus photographs were taken with an auto-focus, auto-shot and auto-tracking fundus camera (Model AFC-230; Nidek, Gamagori, Japan) in an optic disc-centred field of view of 45° in a darkened room. Static retinal vessel analysis (one image of the left or right eye was randomly chosen per participant) was performed using the retinal health information and notification system (RHINO) software developed by the RetinaCheck group of the Technical University of Eindhoven (Eindhoven, the Netherlands) [13, 14]. Optic disc detection and arteriole/venule classification were corrected manually. Retinal vessel diameters were measured at 0.5–1.0 disc diameter away from the optic disc margin and were presented as central retinal arteriolar equivalent and central retinal venular equivalent (CRAE and CRVE, respectively) in measurement units (MU). The scale factor is based on the optic disc diameter, which is assumed to be 1800 μm [15], i.e. 1 MU = 1 pixel size× 1800 μm/pixel size of optic disc diameter. CRAE and CRVE represent the equivalent single-vessel parent diameter for the six largest arterioles and largest venules in the region of interest, respectively. The calculations were based on the improved Knudtson–Parr–Hubbard formula [16].

Fundus photographs of insufficient quality, e.g. obstructed by lashes or defocused, were evaluated and discussed with a second observer and excluded on mutual agreement. We calculated the intraclass correlation coefficients for CRAE and CRVE to assess the agreement between analyses of the RHINO software with vs without manual identification of arterioles and venules using 2556 images. The intraclass correlation coefficient of CRAE was 0.910 and that of CRVE was 0.897.

### Measurement of general characteristics and covariates

History of cardiovascular disease, duration of diabetes, physical activity (h/week), smoking status (never, former, current) and alcohol intake (none/low/high) were assessed by questionnaire [10]. Use of lipid-modifying, antihypertensive and glucose-lowering medication was assessed during a medication interview in which the generic name, dose and frequency were recorded [10]. We measured weight, height, BMI, waist circumference, office and ambulatory 24 h blood pressure, plasma glucose levels, serum creatinine, 24 h urinary albumin excretion (twice), peripheral vibration perception threshold, HbA_1c_ and plasma lipid profile, as described elsewhere [10]. eGFR (in ml min^−1^ 1.73 m^−2^) was calculated with the Chronic Kidney Disease Epidemiology Collaboration equation based on both serum creatinine and serum cystatin C [17]. The presence of retinopathy was assessed in both eyes by use of fundus photographs taken with the same fundus camera (Model AFC-230; Nidek, Gamagori, Japan) as used for measurement of retinal microvascular diameters [10]. Plasma biomarkers of inflammation included high-sensitivity C-reactive protein, serum amyloid A (SAA), IL-6, IL-8 and TNF-α and were measured in EDTA plasma samples with commercially available 4-plex sandwich immunoassay kits (Meso Scale Discovery, Rockville, MD, USA).

### Statistical analysis

Multiple linear regression analysis was used to determine the association of glucose metabolism status (NGM, prediabetes and type 2 diabetes) and measures of blood glucose (HbA_1c_, fasting glucose, 2 h post-load glucose levels) with retinal vessel diameters. For linear trend analyses, the categorical variable glucose metabolism status (NGM = 0, prediabetes = 1, and type 2 diabetes = 2) was used in the regression models. To estimate the difference in retinal microvascular diameters between individuals with prediabetes and type 2 diabetes compared with NGM, we performed analyses with dummy variables for prediabetes and type 2 diabetes. We used the likelihood ratio test to compare models in which glucose metabolism status was treated as a categorical or continuous variable [18]. Model 1 was adjusted for age and sex; Model 2 was additionally adjusted for cardiovascular risk factors that have previously been associated with retinal microvascular diameters (waist circumference, smoking status, office systolic blood pressure, use of antihypertensive and/or lipid-modifying drugs, fasting triacylglycerols and total- to HDL-cholesterol ratio). We also performed a range of additional analyses (see Results for details). A standardised sum score was calculated for plasma markers of inflammation as follows: for each individual biomarker, a *z* score was calculated according to the formula (individual value – population mean)/population standard deviation and the resulting individual biomarker *z* scores were then averaged. A *p* value of <0.05 was considered statistically significant. Interactions of glucose metabolism status and measures of blood glucose with sex and left vs right eye (with regard to the associations between glucose metabolism status with retinal diameters) were tested by incorporating interaction terms (e.g. prediabetes × sex) in the regression models. A *p* for interaction of <0.10 was considered statistically significant. Statistical analyses were performed by use of the Statistical Package for Social Sciences (Version 25.0; IBM, Chicago, IL, USA), except for the likelihood ratio test, which was performed using Stata (Version 14.1; StataCorp, College Station, TX, USA).

## Results

### Characteristics of the study population

Table 1 shows the general characteristics of the study population stratified by glucose metabolism status. The study population consisted of 2876 individuals (98.6% white) with a mean age of 59.8 ± 8.2 years; 51.2% were men, and 28.3% had type 2 diabetes (by design), including both previously diagnosed type 2 diabetes (24.5%) and newly diagnosed type 2 diabetes (3.7%). Individuals with type 2 diabetes and prediabetes, compared with those with NGM, were older (*p*<0.001, ANOVA test), more often male (*p*<0.001, χ^2^ test) and a current smoker (*p*<0.001), and had a higher BMI (*p*<0.001), waist circumference (*p*<0.001), systolic and diastolic blood pressure (*p*<0.001 for both), fasting plasma glucose (*p*<0.001), 2 h post-load glucose (*p*<0.001), HbA_1c_ (*p*<0.001) and triacylglycerol levels (*p*<0.001), lower level of physical activity (*p*<0.001) and lower eGFR (*p*<0.001). The group of individuals with missing data on retinal microvascular measurements or covariates were generally quite similar to those included, but had a higher total- to HDL-cholesterol ratio, more current smokers and insulin use, and smaller CRAE (ESM Table 1).

**Table 1:** General characteristics of the study population according to glucose metabolism status

Characteristics	NGM *n* = 1630	Prediabetes *n* = 433	Type 2 diabetes *n* = 813
Age (years)	57.9 ± 8.1	61.6 ± 7.5	62.6 ± 7.7
Women (*n*, %)	937 (57.5)	202 (46.7)	265 (32.6)
Diabetes duration (years)^a^	–	–	5.0 [1.0–11.0]
BMI (kg/m^2^)	25.5 ± 3.6	27.6 ± 4.2	29.9 ± 5.0
Waist circumference (cm)			
Men	96.2 ± 9.6	102.1 ± 10.3	107.8 ± 12.5
Women	85.8 ± 10.0	93.0 ± 12.7	102.0 ± 14.2
History of cardiovascular disease (*n*, %)	191 (11.7)	56 (12.9)	226 (27.8)
Office SBP (mmHg)	130.6 ± 17.0	137.3 ± 16.8	142.0 ± 18.0
Office DBP (mmHg)	75.2 ± 9.9	77.8 ± 9.4	77.4 ± 9.6
Ambulatory 24 h SBP (mmHg)^b^	117.3 ± 10.9	120.1 ± 11.1	122.4 ± 12.1
Ambulatory 24 h DBP (mmHg)^b^	73.9 ± 7.1	74.5 ± 7.0	73.5 ± 7.3
Hypertension (*n*, %)	659 (40.4)	274 (63.3)	680 (83.6)
Smoking (% never/former/current)	39.8/48.7/11.5	29.1/58.7/12.2	28.9/55.1/16.0
Alcohol intake (% none/low/high)	13.6/59.2/27.3	15.2/54.7/30.0	30.6/51.0/18.3
Physical activity (h/week) ^c^	6.2 ± 4.5	5.2 ± 4.1	4.2 ± 3.9
Fasting glucose (mmol/l)	5.2 ± 0.4	5.9 ± 0.6	7.9 ± 2.0
2 h post-load glucose (mmol/l)^d^	5.4 ± 1.1	8.1 ± 1.7	14.4 ± 3.9
HbA_1c_ (mmol/mol)	35.9 ± 3.7	38.6 ± 4.5	51.9 ± 11.2
HbA_1c_ (%)	5.4 ± 0.3	5.7 ± 0.4	6.9 ± 1.0
Triacylglycerols (mmol/l)	1.2 ± 0.6	1.6 ± 1.0	1.8 ± 1.0
Total- to HDL-cholesterol ratio	3.6 ± 1.1	3.8 ± 1.2	3.7 ± 1.1
Total cholesterol (mmol/l)	5.6 ± 1.0	5.5 ± 1.1	4.4 ± 1.0
HDL (mmol/l)	1.7 ± 0.5	1.5 ± 0.4	1.3 ± 0.4
LDL (mmol/l)	3.4 ± 0.9	3.3 ± 1.0	2.4 ± 0.9
Antihypertensive medication use (*n*, %)	364 (22.3)	199 (46.0)	598 (73.6)
Lipid-modifying medication use (*n*, %)	270 (16.6)	154 (35.6)	609 (74.9)
Diabetes medication use (*n*, %)	0 (0)	0 (0)	641 (78.8)
Insulin	–	–	167 (20.5)
Oral medication only	–	–	600 (73.8)
eGFR (ml min^−1^[1.73 m]^−2^)	90.3 ± 13.1	86.7 ± 14.3	84.8 ± 16.9
Albuminuria (*n*, %) ^e^	66 (4.1)	29 (6.7)	148 (18.4)
Retinopathy (*n*, %)	1 (0.1)	1 (0.2)	34 (4.3)
Neuropathy (*n*,%) ^f^	93 (6.4)	37 (9.9)	147 (20.5)
CRAE (MU)			
Crude	142.9 ± 20.1	141.2 ± 20.2	142.0 ± 21.0
Age- and sex-adjusted	156.0 ± 0.49	156.3 ± 0.94	158.1 ± 0.73
Age-,sex-and office SBP-adjusted	173.1 ± 0.48	174.0 ± 0.93	176.2 ± 0.73
CRVE (MU)			
Crude	213.9 ± 30.7	215.9 ± 31.4	215.6 ± 32.5
Age- and sex-adjusted	223.0 ± 0.75	226.5 ± 1.50	227.2 ± 1.13
Age-, sex- and office SBP-adjusted	228.1 ± 0.75	231.8 ± 1.50	232.6 ± 1.13

Data are reported as mean ± SD or *n* (%) as appropriate, except diabetes duration, which is reported as median [interquartile range], and adjusted CRAE and CRVE which are reported as mean ± SEM

Data present the study population for regression models 1 and 2. SBP, systolic blood pressure; DBP, diastolic blood pressure

^a^Available for 673 individuals with type 2 diabetes

^b^Available for 722 individuals with type 2 diabetes

^c^Available for 685 individuals with type 2 diabetes

^d^Available for 623 individuals with type 2 diabetes

^e^Albuminuria was defined as a urinary albumin excretion of >30 mg per 24 h

^f^Neuropathy was defined as a vibration perception threshold >25 V; data were available for 718 individuals with type 2 diabetes

### Association of glucose metabolism status with retinal microvascular diameters

**Fig. 1:**
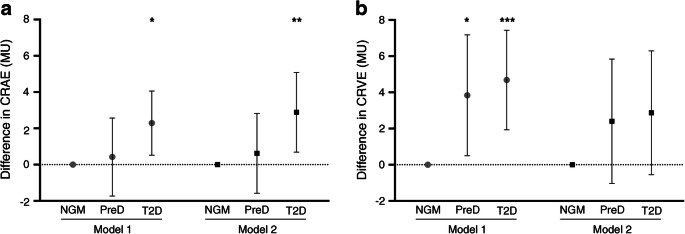
Multivariable-adjusted differences in retinal microvascular diameters between individuals with prediabetes and type 2 diabetes compared with NGM. (**a**) Difference in CRAE. (**b**) Difference in CRVE. Point estimates (β) and 95% CIs represent the difference in retinal microvascular diameters in CRAE and CRVE as compared with NGM. NGM is the reference and is set to zero. Model 1: adjusted for age and sex; Model 2: additional adjustment for waist circumference, smoking status, systolic blood pressure, triacylglycerols, total- to HDL-cholesterol ratio, and use of antihypertensive and/or lipid-modifying drugs. PreD, prediabetes; T2D, type 2 diabetes; MU, measurement unit. *p < 0.05 **p < 0.01 ***p < 0.001 vs NGM in corresponding model

Retinal arterioles were wider (CRAE measured in MU) in individuals with type 2 diabetes compared with those with NGM in the age- and sex-adjusted model (Model 1: β = 2.29 [0.52, 4.06]; Fig. 1a). The association of prediabetes with CRAE was non-significant (Model 1: β = 0.42, [95% CI −1.73, 2.57]; Fig. 1a), whereas CRAE showed a linear trend across glucose metabolism status (Model 1: *p* for trend = 0.013; *p* for likelihood ratio test = 0.49). After further adjustment for cardiovascular risk factors (Model 2), the difference in CRAE between type 2 diabetes and NGM became somewhat larger (prediabetes β = 0.62 [−1.58, 2.83]; type 2 diabetes β = 2.89 [0.69, 5.08]; Fig. 1a). The linear trend for CRAE across glucose metabolism status remained (Model 2: *p* for trend = 0.013; *p* for likelihood ratio test = 0.43).

Retinal venules were wider (CRVE measured in MU) in individuals with prediabetes and type 2 diabetes, compared with those with NGM, in the age- and sex-adjusted model (Model 1: prediabetes β = 3.84 [0.50, 7.18]; type 2 diabetes β = 4.68 [1.93, 7.43]; Fig. 1b), and the CRVE showed a linear trend across glucose metabolism status (Model 1: *p* for trend = 0.001; *p* for likelihood ratio test = 0.36). The difference in CRVE was attenuated and non-significant after adjustment for cardiovascular risk factors (Model 2: prediabetes β = 2.40 [−1.03, 5.84]; type 2 diabetes β = 2.87 [−0.55, 6.29]; Fig. 1b). CRVE no longer showed a linear trend across glucose metabolism status (Model 2: *p* for trend = 0.083; *p* for likelihood ratio test = 0.55).

### Associations of measures of blood glucose with retinal microvascular diameters

**Fig. 2:**
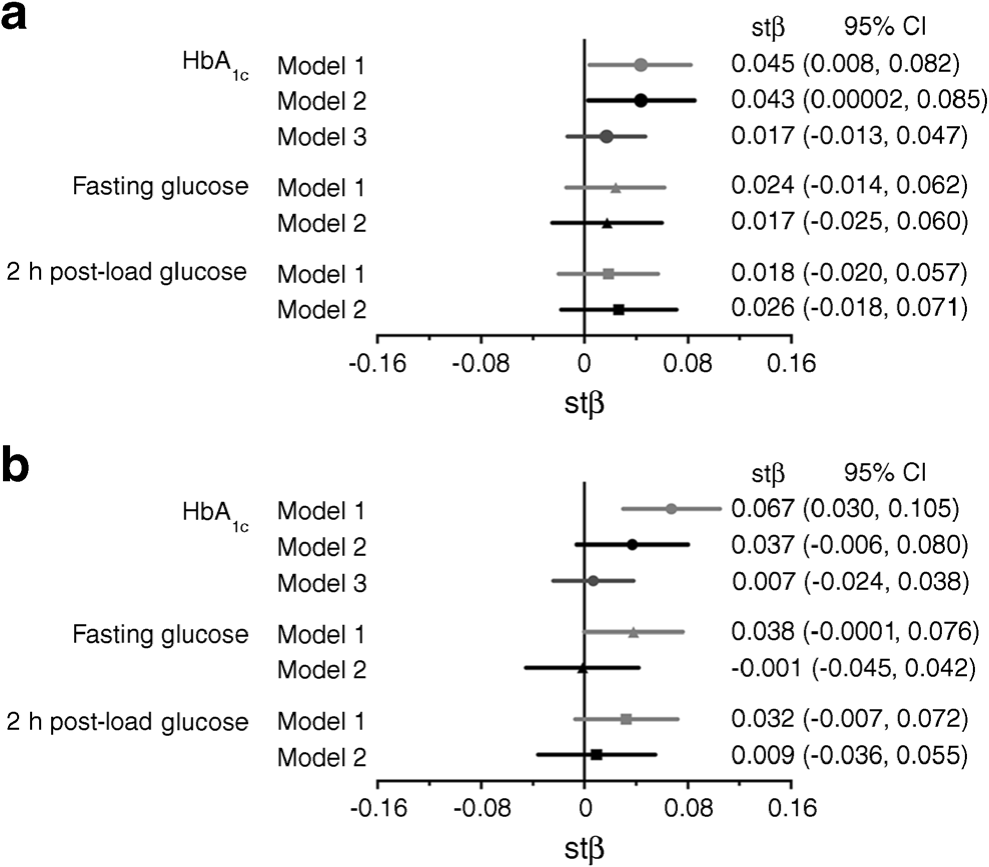
Multivariable-adjusted associations of measures of blood glucose with retinal microvascular diameters. (**a**) Associations of measures of blood glucose with CRAE. (**b**) Associations of measures of blood glucose with CRVE. Point estimates (standardised β [stβ]) and 95% CIs represent the difference (in SD) in retinal microvascular diameters per SD increase in the measure of blood glucose. Model 1: adjusted for age and sex; Model 2: additional adjustment for waist circumference, smoking status, systolic blood pressure, triacylglycerols, total- to HDL-cholesterol ratio, and use of antihypertensive and/or lipid-modifying drugs. Model 3: additional adjustment for CRVE in models of CRAE and adjustment for CRAE in models of CRVE

**Fig. 3:**
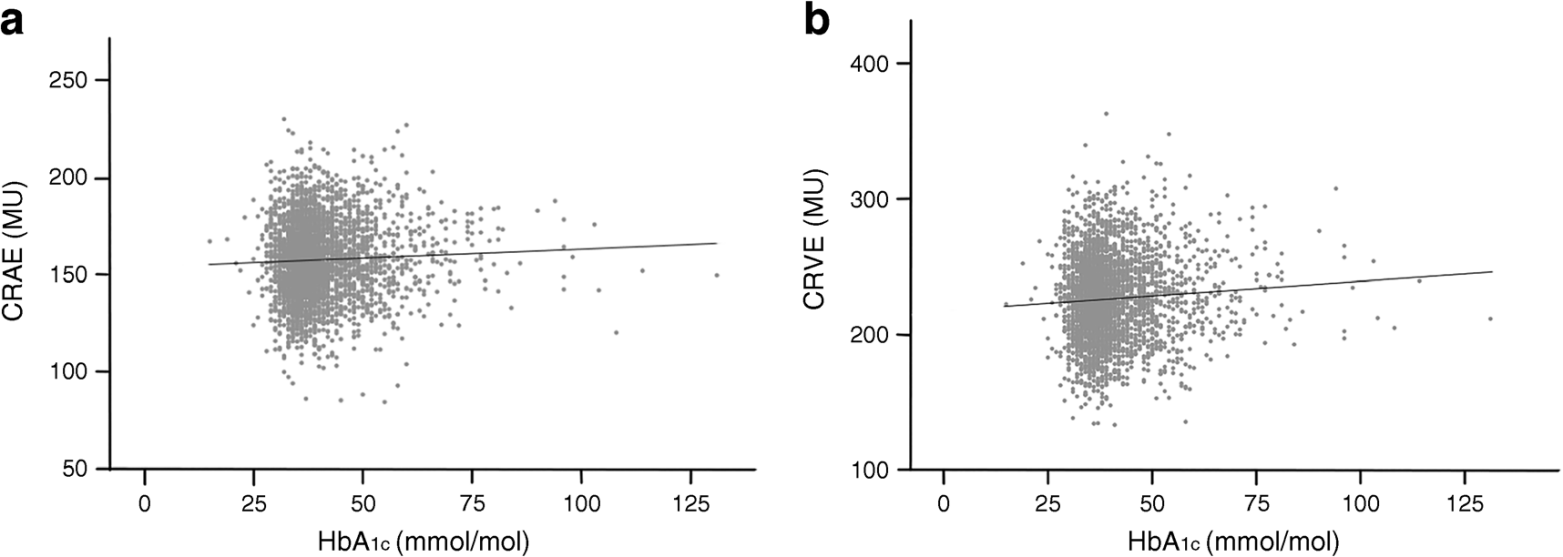
Age- and sex-adjusted association of HbA_1c_ with retinal microvascular diameters. (**a**) Association between HbA_1c_ and CRAE (β = 0.09 [95% CI 0.02, 0.17]); (**b)** Association between HbA_1c_ and CRVE (β = 0.22 [0.10, 0.34]). Regression coefficients (β) indicate the age- and sex-adjusted mean difference and 95% CI in CRAE and CRVE per 1 mmol/mol increase in HbA_1c_

Higher levels of HbA_1c_ were associated with greater CRAE after adjustment for age and sex (Model 1; Figs. 2a and 3a), and also after further adjustment for cardiovascular risk factors (Model 2; Fig. 2a). Higher levels of fasting glucose and 2 h post-load glucose were not statistically significantly associated with greater CRAE (Fig. 2a).

Higher levels of HbA_1c_ were associated with greater CRVE after adjustment for age and sex (Model 1; Figs. 2b and 3b). The association was attenuated and non-significant after further adjustment for cardiovascular risk factors (Model 2; Fig. 2b). Higher fasting glucose and 2 h post-load glucose were not statistically significantly associated with greater CRVE (Fig. 2b).

### Additional analyses

Further analyses to assess the robustness of our observations are described in the ESM results (ESM Tables 2–7); in general, these analyses confirmed the observations reported above. To explore whether retinal diameters are intrinsically linked, we analysed venular diameters as a function of arteriolar diameters. We found that retinal arteriolar diameters were positively associated with retinal venular diameters after adjustment for age and sex (β = 1.09 [1.05, 1.13], *p* < 0.001; ESM Fig. 2). The association remained similar after further adjustment for height, body surface area, systolic blood pressure and HbA_1c_ level (β = 1.09 [1.05, 1.13], *p* < 0.001). In addition, to explore whether the associations of glucose metabolism status and measures of blood glucose with CRAE and CRVE are linked, we additionally adjusted for CRVE in models of CRAE and for CRAE in models of CRVE. We found that further adjustment for CRVE attenuated the difference in CRAE in prediabetes and type 2 diabetes vs NGM (prediabetes β = −0.46 [−2.03, 1.11]; type 2 diabetes β = 1.60 [0.03, 3.16]; *p* for trend = 0.073), while further adjustment for CRAE completely eliminated the difference in CRVE (prediabetes β = 1.72 [−0.73 to 4.18]; type 2 diabetes β = −0.28 [−2.73, 2.16]; *p* for trend = 0.984). Similarly, the association of HbA_1c_ with CRAE was attenuated after further adjustment for CRVE, while the association with CRVE was eliminated after further adjustment for CRAE (Model 3; Fig. 2).

We did not find any significant associations between duration of type 2 diabetes and retinal microvascular diameters (available for *n* = 673 individuals; ESM Table 8).

## Discussion

This study shows that type 2 diabetes, higher levels of HbA_1c_ and, possibly, prediabetes are associated with wider retinal arteriolar diameters in a predominantly white population. Notably, the associations with retinal arteriolar diameters are independent of a broad array of potential confounders. These findings indicate that retinal microvascular changes already occur prior to the diagnosis of type 2 diabetes. In addition, retinal arteriolar diameters are associated with retinal venular diameters, independently of age, sex, height, body surface area, blood pressure and blood glucose, which suggests a close link between arteriolar and venular dilation in general and, thus, also in (pre)diabetes.

Our results indicate that type 2 diabetes and, possibly, prediabetes are independently associated with wider retinal arteriolar diameters, which is consistent with previous cross-sectional studies [3–9]. Compared with these studies, we used OGTT and HbA_1c_, which are more accurate measurements for classifing glucose metabolism status than measuring fasting glucose, random glucose, and HbA_1c_ levels only [19]. In addition, we showed that the associations were independent of a broad array of cardiovascular risk factors. Notably, we found that age, sex and systolic blood pressure had strong confounding effects. For example, older age, male sex and higher blood pressure were associated with both narrower arterioles and (pre)diabetes. Unsurprisingly, eliminating these confounders through statistical adjustment reversed the direction of association between (pre)diabetes and retinal arteriolar diameters (Table 1). Note also that we included ambulatory 24 h blood pressure as a confounder in our additional analyses, as it is more accurate than office blood pressure [20] and has not been used in previous studies [3–9], making residual confounding by inaccurately measured blood pressure much less likely in our study. With regard to the outcomes, we measured diameters with semi-automated software (RHINO), which was validated manually and had a relative error that was comparable to that of Interactive Vessel Analysis (IVAN) software [21]. Finally, we used linear trend analyses, as we hypothesised that the difference in retinal microvascular diameters from NGM to prediabetes to type 2 diabetes is of a continuous nature. The results of these analyses favour the interpretation that arteriolar widening occurs in both type 2 diabetes and prediabetes. In support of this, HbA_1c_, a continuous measure of blood glucose, was significantly associated with retinal arteriolar diameters. Although we cannot exclude the possibility that there is no true association between prediabetes and greater arteriolar diameter, we attribute the lack of statistical significance of the difference between prediabetes and NGM with regard to retinal arteriolar diameters to a type 2 statistical error, because the power of between-group comparisons was reduced compared with the power of trend analyses.

Retinal arteriolar dilation in (pre)diabetes is thought to be a result of impaired arteriolar autoregulation [22]. Lacking neuronal innervation, retinal arterioles are affected mainly by local autoregulation through the release of vasoactive substances by microvascular endothelium and the myogenic response of smooth muscle cells [23]. For example, in retinal arteriolar smooth muscle cells, hyperglycaemia and hypoxia can cause endothelin-1 resistance and inhibit Ca^2+^ influx channels [24, 25]. In addition, death and insufficient renewal of endothelial cells, smooth muscle cells and pericytes can further weaken arteriolar wall and boost dilation.

The associations of prediabetes and type 2 diabetes with retinal venular diameters were directionally similar to those for arterioles even though they were not statistically significant after adjustment for cardiovascular risk factors. The non-significance of the associations may be explained by four factors. First, the relatively larger measurement error of venular vs arteriolar diameters [21] decreases the precision of the association with blood glucose and thus increases the confidence interval [26]. Second, our additional analyses (Fig. 2 and ESM Fig. 2) are consistent with the concept that (glucose-related) arteriolar widening drives venular widening to an important extent, possibly by greater transmission of blood pressure. Such mediation will tend to bias the association between blood glucose and venular diameters towards the null [27]. Third, longitudinal studies [28–30] have suggested that widening of retinal venules may also occur before prediabetes, which may reduce the difference in venular diameter between (pre)diabetes and NGM. Fourth, our fully adjusted model may have been over-adjusted as a result of the inclusion of waist circumference, since obesity may be on the causal pathway between (pre)diabetes and retinal venular dilation [31].

In general, studies on the associations between diabetes or blood glucose and retinal venular diameters have not shown consistent results [3–9], although venular widening has been much more consistently observed among Asian populations [3, 4, 6–8] than among white populations [3, 5, 9]. These inconsistent results may be attributed to ethnicity but also to differences in classification of glucose metabolism status, insufficient adjustment for confounding and different types of software used.

The pathophysiological mechanisms that explain retinal venular dilation in (pre)diabetes remain unclear. Retinal venules have been proposed to dilate in response to diabetes-associated inflammation [3, 32], but the association was unchanged after adjustment for inflammation in our study and a previous study [3]. As alluded to above (Fig. 2 and ESM Fig. 2), our results are consistent with the hypothesis that retinal venular dilation is, at least in part, a direct consequence of arteriolar dysfunction [24]. However, we cannot exclude the possibility that, alternatively or additionally, wider venules could lead to wider arterioles, for example, through arteriole to venule shunting and local tissue hypoxia.

Retinal arteriolar dilation is associated with progression of retinopathy [33] and presence of neuropathy [34]. Retinal venular dilation is similarly associated with incidence and progression of retinopathy [35], incidence of nephropathy [36], prevalence and incidence of stroke [37, 38] and progression of cerebral small vessel disease [39]. Taken together with our findings, these results may explain why such complications are commonly present at diagnosis of type 2 diabetes or sometimes before. Retinal microvascular dilation seems to be reversible [40–42]; however, whether this improvement in retinal microvascular dilation will translate into an improved prognosis with respect to complications of diabetes needs further investigation.

Strengths of our study include the population-based design with oversampling of individuals with type 2 diabetes; the use of OGTT to characterise glucose metabolism status; the extensive phenotyping, which enables detection of independent associations after extensive adjustments for potential confounders; and the broad array of additional analyses, which gave deeper insight into the associations. Our study also has limitations. First, the cross-sectional data cannot definitively establish a causal link between (pre)diabetes and retinal microvascular features. Nevertheless, there is extensive evidence that hyperglycaemia causes microvascular dysfunction, and that the association may in fact be bidirectional [43, 44]. Second, our study population was 40–75 years of age, predominantly white, with relatively well-controlled blood glucose and cardiovascular risk factors, which should be taken into consideration when the findings are extrapolated to other populations. Third, although treating glucose metabolism status as a continuous variable increased statistical power to detect the associations of (pre)diabetes with retinal microvascular diameters and the results of likelihood ratio test confirmed the feasibility of this approach, it may also introduce bias into the estimates of associations, which are largely influenced by the difference in retinal microvascular diameters between the two extreme groups, i.e. the NGM and type 2 diabetes groups. Fourth, our fully adjusted model may have been over-adjusted, as a result of the inclusion of waist circumference, since for retinal venular diameters, and therefore the association of (pre)diabetes with retinal microvascular diameters may have been underestimated. Fifth, although investigational procedures were standardised, participants were allowed a light meal, which will increase variation in retinal microvacular diameters and thus bias associations towards the null. Sixth, although we adjusted for major potential confounders, there is still a possibility of residual confounding by variables that were not included in the analyses.

In summary, this study has demonstrated that type 2 diabetes, higher levels of HbA_1c_, and, possibly, prediabetes are associated with wider retinal arterioles, independent of major cardiovascular risk factors, in a predominantly white population. These results support the ‘ticking clock’ hypothesis, which postulates that microvascular dysfunction precedes the clinical diagnosis of type 2 diabetes [43, 44], and may partly explain the occurrence of complications related to microvascular dysfunction in prediabetes and in early type 2 diabetes. Thus, microvascular dysfunction can be considered an early marker of (pre)diabetes and a potential target for intervention.

## Electronic supplementary material

ESM 1 (PDF 2.15 mb)

## Data Availability

The datasets generated during and/or analysed during the current study are available from the corresponding author on reasonable request.

## Abbreviations

CRAE: Central retinal arteriolar equivalent
CRVE: Central retinal venular equivalent
MU: Measurement unit
NGM: Normal glucose metabolism
RHINO: Retinal health information and notification system
