# Supplementary material for: Type 2 diabetes and HbA1c are independently associated with wider retinal arterioles: the Maastricht study
Source: Diabetologia. 2020 May 8;63(7):1408–17. doi: 10.1007/s00125-020-05146-z (PMC7286946; doi:10.1007/s00125-020-05146-z)
Supplement: Supplementary file 1 — (PDF 2.15 mb) [file 125_2020_5146_MOESM1_ESM.pdf]

ESM results

Additional analyses

Associations remained similar when we replaced office systolic pressure with 24 h ambulatory systolic blood pressure (available in 2545 individuals； ESM Table 2), or further specified blood pressure-lowering medication into renin-angiotensin-aldosterone system (RAAS) inhibitors and other types of antihypertensives (available in 2876 individuals; ESM Table 3) in the regression models. Associations also remained similar when we replaced waist circumference with body mass index (BMI; available in 2875 individuals; ESM Table 3).

Associations again remained similar after additional adjustment for presence of retinopathy, presence of neuropathy, eGFR and albumin excretion, or history of cardiovascular disease (available in 2328 individuals; ESM Table 4). The associations also remained when physical activity and alcohol intake (available in 2535 individuals, ESM Table 5) were added to the models. Additional adjustment for plasma markers of inflammation (CRP, SAA, IL-6, IL-8, and TNF-α) and their standardised sum score did not materially change the associations (available in 2848 individuals; ESM Table 6).

Associations also remained similar when we excluded participants with outliers (defined as <3SD or >3SD) in retinal microvascular diameters (*n*=18; ESM Table 7), excluded participants with retinopathy (*n*=36; ESM Table 7), excluded participants with catch-up fundus photography (*n*=176; ESM Table 7),

or excluded non-white population (*n*=41; ESM Table 7). Finally, we did not find any significant interactions with sex (*p* for interaction >0.440) or left versus right eye (*p* for interaction >0.579) with regard to the associations between glucose metabolism variables on the one hand and retinal outcomes on the other.

ESM tables

ESM Table 1: General characteristics of the study population and individuals excluded from the analyses due to missing values

Characteristic	Study population (N =2876)	Missing	Excluded due to missing values (N =534)	*p*-value
Age (years)	59.8 ± 8.2	0	59.9 ± 8.6	0.736
Women (n,%)	1404 (48.8)	0	250 (46.8)	0.397
Glucose metabolism status (n,%)		0		0.624
- Normal glucose metabolism	1630 (56.7)	0	294 (55.1)	
- Prediabetes	433 (15.1)	0	78 (14.6)	
- Type 2 diabetes	813 (28.3)	0	162 (30.3)	
Diabetes duration (years) [a]	5.0 [1.0-11.0]	177	5.0 [1.5-11.0]	0.601
Body mass index (kg/m^2)	27.1 ± 4.5	3	27.4 ± 4.8	0.166
Waist circumference (cm)		4		
- Men	101.5 ± 12.1	2	102.2 ± 12.5	0.340
- Women	89.9 ± 12.9	2	91.0 ± 13.5	0.234
History of cardiovascular disease (n,%)	473 (16.6)	65	88 (18.0)	0.431
Office SBP (mmHg)	134.8 ± 18.0	2	136.1 ± 19.2	0.174
Office DBP (mmHg)	76.2 ± 9.8	2	76.0 ± 9.8	0.678
Ambulatory 24 h SBP (mmHg) [b]	119.2 ± 11.5	416	119.6 ± 13.1	0.507
Ambulatory 24 h DBP (mmHg) [b]	73.9 ± 7.2	416	74.1 ± 7.1	0.561
Hypertension (n,%)	1613 (56.1)	6	302 (56.9)	0.775
Physical activity (hours/week) [c]	5.5 ± 4.3	432	5.8 ± 4.7	0.136

Smoking (%never/former/current)	35.1/52.0/12.9	49	31.5/49.9/18.6	0.003
Alcohol intake (%none/low/high)	18.6/56.2/25.2	47	19.4/51.3/29.2	0.099
Fasting glucose (mmol/l)	6.1 ± 1.6	1	6.1 ± 1.8	0.285
2 h postload glucose (mmol/l) [d]	7.9 ± 4.3	251	7.7 ± 4.0	0.168
HbA_{1c} (mmol/mol)	40.8 ± 9.8	13	42.5 ± 10.8	<0.001
HbA_{1c} (%)	5.9 ± 0.9	13	6.0 ± 1.0	<0.001
Triglycerides (mmol/l)	1.4 ± 0.8	4	1.5 ± 0.9	0.243
Total-to-HDL cholesterol ratio	3.6 ± 1.2	4	4.0 ± 1.2	<0.001
Total cholesterol (mmol/l)	5.2 ± 1.2	4	5.2 ± 1.2	0.701
HDL cholesterol (mmol/l)	1.5 ± 0.5	4	1.4 ± 0.4	<0.001
LDL cholesterol (mmol/l)	3.1 ± 1.0	4	3.2 ± 1.0	0.026
Antihypertensive medication use (n,%)	1161 (40.4)	0	208 (39.0)	0.564
Lipid-modifying medication use (n,%)	1033 (35.9)	0	198 (37.1)	0.624
Diabetes medication use (n,%)	641 (22.3)	0	125 (23.4)	0.573
- Insulin	167 (5.8)	0	49 (9.2)	0.005
- Oral medication only	600 (20.9)	0	114 (21.3)	0.817
eGFR (ml/min/1.73m^2)	88.2 ± 14.7	33	87.5 ± 15.9	0.314
Albuminuria (n,%) [e]	243 (8.5)	42	48 (9.4)	0.732
Retinopathy (n,%)	36 (1.3)	587	4 (3.0)	0.114
Neuropathy (n,%) [f]	277 (10.9)	470	49 (12.2)	0.441
CRAE (MU)	142.3 ± 20.4	486	136.4 ± 21.5	0.045
CRVE (MU)	214.7 ± 31.3	486	214.0 ± 33.3	0.875

Data are reported as mean ± SD or number (percentages %) as appropriate, except diabetes duration, which is reported as median [interquartile range]. Student's t and chi-square tests were used to compare continuous and categorical variables between the study population and individuals excluded due to missing values, respectively. The Mann-Whitney test was used to compare diabetes duration between the two populations. SBP, systolic blood pressure; DBP, diastolic blood pressure; HbA_{1c}, hemoglobin A1c; HDL,

high density lipoprotein; LDL, low density lipoprotein; eGFR, estimated glomerular filtration rate; CRAE, central retinal arteriolar equivalent; CRVE, central retinal venular equivalent; MU, measurement unit.

[a] Available in 673 in the study population and 125 in the excluded group

[b] Available in 2545 in the study population and 449 in the excluded group

[c] Available in 2535 in the study population and 443 in the excluded group

[d] Available in 2685 in the study population and 474 in the excluded group

[e] Albuminuria was defined as a urinary albumin excretion of > 30 mg per 24 hours

[f] Neuropathy was defined as a vibration perception threshold >25 V, and data were available in 2539 in the study population and 401 in the excluded group.

ESM Table 2: Multivariable-adjusted difference in retinal vascular diameters in individuals with prediabetes and type 2 diabetes versus normal glucose metabolism (NGM) with replacement of office by 24 h systolic ambulatory blood pressure in regression models

**Characteristic**	**Prediabetes [a] (β (95% CI))**	**Type 2 diabetes [a] (β (95% CI))**	***p* for Trend**
CRAE (MU)			
Model 1	0.02 (-2.29 to 2.32)	1.67 (-0.21 to 3.55)	0.096
Model 2a	0.12 (-2.24 to 2.48)	2.27 (-0.05 to 4.58)	0.069
Model 2b	0.03 (-2.33 to 2.38)	2.09 (-0.22 to 4.39)	0.094
CRVE (MU)			
Model 1	3.47 (-0.12 to 7.05)	4.43 (1.50 to 7.36)	0.002
Model 2a	2.03 (-1.65 to 5.71)	2.93 (-0.67 to 6.53)	0.099
Model 2b	1.98 (-1.69 to 5.66)	2.83 (-0.76 to 6.43)	0.110

Regression coefficients (β) indicate the mean difference (95% CI) in retinal microvascular diameters with normal glucose metabolism as reference. CI: confidence interval. CRAE: Central retinal arteriolar equivalent. CRVE: Central retinal venular equivalent. MU: Measurement unit. [a] 24-h systolic ambulatory blood pressure was available in n=2545 of the study population (375 individuals with prediabetes and 722 individuals with type 2 diabetes).

Model 1: adjustment for age and sex

Model 2a: Model 1+adjustment for waist circumference, triglyceride levels, total-to-high-density-lipoprotein ratio, smoking status, office systolic blood pressure, use of antihypertensive medication, and use of lipid-modifying medication

Model 2b: Model 1+adjustment for waist circumference, triglyceride levels, total-to-high-density-lipoprotein ratio, smoking status, 24 h systolic ambulatory blood pressure, use of antihypertensive medication, and use of lipid-modifying medication

ESM Table 3: Multivariable-adjusted difference in retinal vascular diameters in individuals with prediabetes and type 2 diabetes versus normal glucose metabolism (NGM) with adjustment for renin-angiotensin-aldosterone system (RAAS)-inhibiting antihypertensives and other types of antihypertensives or replacement of waist circumference by body mass index (BMI) in regression models

Characteristic	Prediabetes (β (95% CI))	Type 2 diabetes (β (95% CI))	*p* for Trend
CRAE (MU)			
Model 1	0.42 (-1.73 to 2.57)	2.29 (0.52 to 4.06)	0.013
Model 2a	0.62 (-1.58 to 2.83)	2.89 (0.69 to 5.08)	0.013
Model 2b	0.60 (-1.60 to 2.80)	2.83 (0.64 to 5.03)	0.014
Model 2c	0.64 (-1.57 to 2.84)	2.86 (0.67 to 5.06)	0.013
Model 2d	0.53 (-1.67 to 2.73)	2.69 (0.52 to 4.87)	0.019
CRVE (MU)			
Model 1	3.84 (0.50 to 7.18)	4.68 (1.93 to 7.43)	0.001
Model 2a	2.40 (-1.03 to 5.84)	2.87 (-0.55 to 6.29)	0.083
Model 2b	2.37 (-1.06 to 5.80)	2.89 (-0.53 to 6.31)	0.081
Model 2c	2.38 (-1.06 to 5.82)	2.90 (-0.53 to 6.32)	0.080
Model 2d	2.39 (-1.04 to 5.82)	2.87 (-0.52 to 6.25)	0.080

Regression coefficients (β) indicate the mean difference (95% CI) in retinal microvascular diameters with normal glucose metabolism as reference. CI: confidence interval. CRAE: Central retinal arteriolar equivalent. CRVE: Central retinal venular equivalent. MU: Measurement unit. RAAS-inhibiting antihypertensives included angiotensin-converting-enzyme inhibitors, angiotensin receptor blockers, and renin blockers.

Model 1: adjustment for age and sex

Model 2a: Model 1+adjustment for waist circumference, triglyceride levels, total-to-high-density-lipoprotein ratio, smoking status, office systolic blood pressure, use of antihypertensive medication, and use of lipid-modifying medication

Model 2b: Model 1+adjustment for waist circumference, triglyceride levels, total-to-high-density-lipoprotein ratio, smoking status, office systolic blood pressure, use of RAAS-inhibiting antihypertensives, and use of lipid-modifying medication

Model 2c: Model 1+adjustment for waist circumference, triglyceride levels, total-to-high-density-lipoprotein ratio, smoking status, office systolic blood pressure, use of RAAS-inhibiting antihypertensives, use of non-RAAS-inhibiting antihypertensives and use of lipid-modifying medication

Model 2d: Model 1+adjustment for BMI, triglyceride levels, total-to-high-density-lipoprotein ratio, smoking status, office systolic blood pressure, use of antihypertensive medication, and use of lipid-modifying medication

ESM Table 4: Multivariable-adjusted difference in retinal vascular diameters in individuals with prediabetes and type 2 diabetes versus normal glucose metabolism (NGM) with additional adjustment for presence of retinopathy, presence of neuropathy, estimated glomerular filtration rate (eGFR) and albumin excretion, and history of cardiovascular disease

**Characteristic**	**Prediabetes [a] (β (95% CI))**	**Type 2 diabetes [a] (β (95% CI))**	***p* for Trend**
CRAE (MU)			
Model 1	1.85 (-0.52 to 4.23)	1.92 (-0.002 to 3.84)	0.040
Model 2a	2.16 (-0.26 to 4.59)	2.40 (0.05 to 4.76)	0.034
Model 2b	2.17 (-0.26 to 4.59)	2.37 (0.001 to 4.74)	0.036
Model 2c	2.16 (-0.26 to 4.59)	2.31 (-0.07 to 4.69)	0.042
Model 2d	2.41 (-0.01 to 4.82)	2.51 (0.14 to 4.89)	0.026
Model 2e	2.51 (0.09 to 4.93)	2.54 (0.16 to 4.92)	0.024
CRVE (MU)			
Model 1	4.41 (0.70 to 8.13)	4.80 (1.80 to 7.81)	0.001
Model 2a	3.31 (-0.49 to 7.11)	3.10 (-0.60 to 6.80)	0.075
Model 2b	3.33 (-0.47 to 7.13)	2.89 (-0.83 to 6.61)	0.094
Model 2c	3.33 (-0.48 to 7.13)	2.88 (-0.86 to 6.61)	0.096
Model 2d	3.75 (-0.05 to 7.54)	3.17 (-0.55 to 6.90)	0.065
Model 2e	3.89 (0.09 to 7.69)	3.21 (-0.52 to 6.94)	0.061

Regression coefficients (β) indicate the mean difference (95% CI) in retinal microvascular diameters with normal glucose metabolism as reference. CI: confidence interval. CRAE: Central retinal arteriolar equivalent. CRVE: Central retinal venular equivalent. MU: Measurement unit. [a] Data were available in n=2328 of the study population (344 individuals with prediabetes and 683 individuals with type 2 diabetes).

Model 1: adjustment for age and sex

Model 2a: Model 1+adjustment for waist circumference, triglyceride levels, total-to-high-density-lipoprotein ratio, smoking status, office systolic blood pressure, use of antihypertensive medication, and use of lipid-modifying medication

Model 2b: Model 2a+adjustment for retinopathy
Model 2c: Model 2b+adjustment for neuropathy (vibration perception thresholds >25 V)
Model 2d: Model 2c+adjustment for eGFR and albumin excretion
Model 2e: Model 2d+adjustment for history of cardiovascular disease

ESM Table 5: Multivariable-adjusted difference in retinal vascular diameters in individuals with prediabetes and type 2 diabetes versus normal glucose metabolism (NGM) with additional adjustment for physical activity and alcohol intake

Characteristic	Prediabetes [a] (β (95% CI))	Type 2 diabetes [a] (β (95% CI))	*p* for Trend
CRAE (MU)			
Model 1	0.46 (-1.78 to 2.70)	1.92 (0.05 to 3.79)	0.049
Model 2a	0.72 (-1.58 to 3.02)	2.63 (0.32 to 4.95)	0.030
Model 2b	0.71 (-1.59 to 3.01)	2.62 (0.30 to 4.94)	0.031
Model 2c	0.66 (-1.63 to 2.96)	2.17 (-0.18 to 4.52)	0.076
CRVE (MU)			
Model 1	3.94 (0.43 to 7.45)	3.39 (0.46 to 6.33)	0.014
Model 2a	2.53 (-1.08 to 6.15)	1.98 (-1.67 to 5.63)	0.226
Model 2b	2.53 (-1.09 to 6.15)	1.99 (-1.67 to 5.64)	0.227
Model 2c	2.50 (-1.12 to 6.12)	1.68 (-2.01 to 5.38)	0.293

Regression coefficients (β) indicate the mean difference (95% CI) in retinal microvascular diameters with normal glucose metabolism as reference. CI: confidence interval. CRAE: Central retinal arteriolar equivalent. CRVE: Central retinal venular equivalent. MU: Measurement unit. [a] Data were available in n=2535 of the study population (387 individuals with prediabetes and 685 individuals with type 2 diabetes).

Model 1: adjustment for age and sex

Model 2a: Model 1+adjustment for waist circumference, triglyceride levels, total-to-high-density-lipoprotein ratio, smoking status, office systolic blood pressure, use of antihypertensive medication, and use of lipid-modifying medication

Model 2b: Model 2a+adjustment for physical activity

Model 2c: Model 2b+adjustment for alcohol intake

ESM Table 6: Multivariable-adjusted difference in retinal vascular diameters in individuals with prediabetes and type 2 diabetes versus normal glucose metabolism (NGM) with additional adjustment for inflammation

**Characteristic**	**Prediabetes [a] (β (95% CI))**	**Type 2 diabetes [a] (β (95% CI))**	***p* for Trend**
CRAE (MU)			
Model 1	0.32 (-1.83 to 2.48)	2.29 (0.51 to 4.07)	0.014
Model 2a	0.55 (-1.66 to 2.76)	2.88 (0.68 to 5.09)	0.013
Model 2b	0.54 (-1.67 to 2.76)	2.87 (0.67 to 5.08)	0.014
CRVE (MU)			
Model 1	3.53 (0.19 to 6.87)	4.77 (2.01 to 7.52)	<0.001
Model 2a	2.17 (-1.27 to 5.61)	3.01 (-0.42 to 6.44)	0.074
Model 2b	2.06 (-1.38 to 5.50)	2.87 (-0.57 to 6.31)	0.089

Regression coefficients (β) indicate the mean difference (95% CI) in retinal microvascular diameters with normal glucose metabolism as reference. CI: confidence interval. CRAE: Central retinal arteriolar equivalent. CRVE: Central retinal venular equivalent. MU: Measurement unit. [a] Inflammation was available in n=2848 of the study population (430 individuals with prediabetes and 803 individuals with type 2 diabetes).

Model 1: adjustment for age and sex

Model 2a: Model 1+adjustment for waist circumference, triglyceride levels, total-to-high-density-lipoprotein ratio, smoking status, office systolic blood pressure, use of antihypertensive medication, and use of lipid-modifying medication

Model 2b: Model 2+adjustment for sum score of inflammation

ESM Table 7: Multivariable-adjusted difference in retinal vascular diameters in individuals with prediabetes and type 2 diabetes versus normal glucose metabolism (NGM) excluding outliers in retinal microvascular diameters, participants with retinopathy, participants with catch-up fundus photography, or non-white population

Characteristic	Prediabetes (β (95% CI))	Type 2 diabetes (β (95% CI))	*p* for Trend
Excluding outliers (defined as <3SD or >3SD) in retinal microvascular diameters (n=18)			
CRAE (MU)			
Model 1	0.74 (-1.37 to 2.84)	2.50 (0.77 to 4.24)	0.005
Model 2	0.87 (-1.29 to 3.03)	3.00 (0.84 to 5.15)	0.008
CRVE (MU)			
Model 1	3.89 (0.60 to 7.18)	4.99 (2.28 to 7.71)	<0.001
Model 2	2.47 (-0.91 to 5.86)	3.28 (-0.09 to 6.66)	0.046
Excluding participants with retinopathy (n=36)			
CRAE (MU)			
Model 1	0.41 (-1.75 to 2.56)	2.27 (0.47 to 4.06)	0.016
Model 2	0.59 (-1.62 to 2.80)	2.80 (0.58 to 5.02)	0.017
CRVE (MU)			
Model 1	3.77 (0.42 to 7.11)	4.43 (1.65 to 7.22)	0.001
Model 2	2.28 (-1.16 to 5.72)	2.53 (-0.93 to 5.98)	0.125
Excluding participants with catch-up fundus photography (n=176)			
CRAE (MU)			
Model 1	0.56 (-1.65 to 2.77)	2.41 (0.61 to 4.22)	0.010
Model 2	0.78 (-1.49 to 3.04)	3.04 (0.81 to 5.27)	0.009
CRVE (MU)			
Model 1	3.67 (0.22 to 7.13)	4.80 (1.98 to 7.62)	0.001
Model 2	2.26 (-1.29 to 5.82)	2.99 (-0.52 to 6.49)	0.082

Excluding non-white population (n=41)			
CRAE (MU)			
Model 1	0.43 (-1.73 to 2.59)	2.24 (0.45 to 4.03)	0.017
Model 2	0.65 (-1.57 to 2.86)	2.91 (0.69 to 5.13)	0.013
CRVE (MU)			
Model 1	3.74 (0.39 to 7.10)	4.32 (1.54 to 7.10)	0.001
Model 2	2.39 (-1.06 to 5.84)	2.62 (-0.84 to 6.08)	0.112

Regression coefficients (β) indicate the mean difference (95% CI) in retinal microvascular diameters with normal glucose metabolism as reference. CI: confidence interval. CRAE: Central retinal arteriolar equivalent. CRVE: Central retinal venular equivalent. MU: Measurement unit.

Model 1: adjustment for age and sex

Model 2: additional adjustment for waist circumference, triglyceride levels, total-to-high-density-lipoprotein ratio, smoking status, office systolic blood pressure, use of antihypertensive medication, and use of lipid-modifying medication

ESM Table 8: Multivariable-adjusted association between duration of type 2 diabetes and retinal microvascular diameters

**Characteristic**	**Duration of type 2 diabetes**		***p* for trend**
	**2nd tertile β (95% CI)**	**3rd tertile β (95% CI)**	
CRAE (MU)			
Model 1	-1.59 (-5.11 to 1.93)	0.09 (-3.43 to 3.61)	0.711
Model 2	-0.95 (-4.53 to 2.62)	1.43 (-2.20 to 5.06)	0.315
CRVE (MU)			
Model 1	-3.60 (-9.08 to 1.88)	-4.01 (-9.50 to 1.47)	0.441
Model 2	-2.48 (-7.99 to 3.02)	-2.09 (-7.69 to 3.51)	0.868

Regression coefficients (β) indicate the mean difference (95% CI) in retinal microvascular diameters with the lowest tertile as reference. CI: confidence interval. CRAE: Central retinal arteriolar equivalent. CRVE: Central retinal venular equivalent. MU: Measurement unit. Data were available in 673 individuals.

Model 1: adjustment for age and sex

Model 2: Model 1+adjustment for waist circumference, triglyceride levels, total-to-high-density-lipoprotein ratio, smoking status, office systolic blood pressure, use of antihypertensive medication, and use of lipid-modifying medication

ESM figures

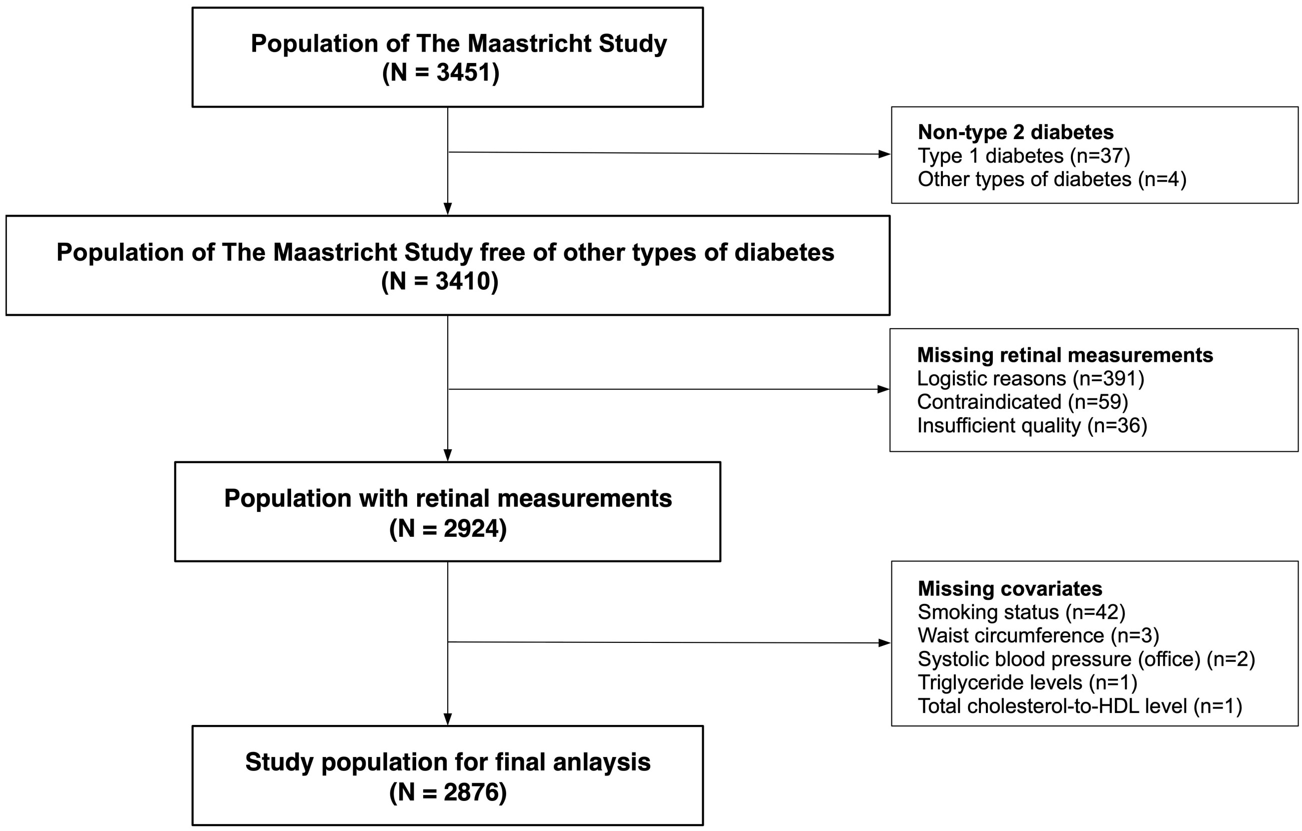

ESM Figure 1. Study population selection

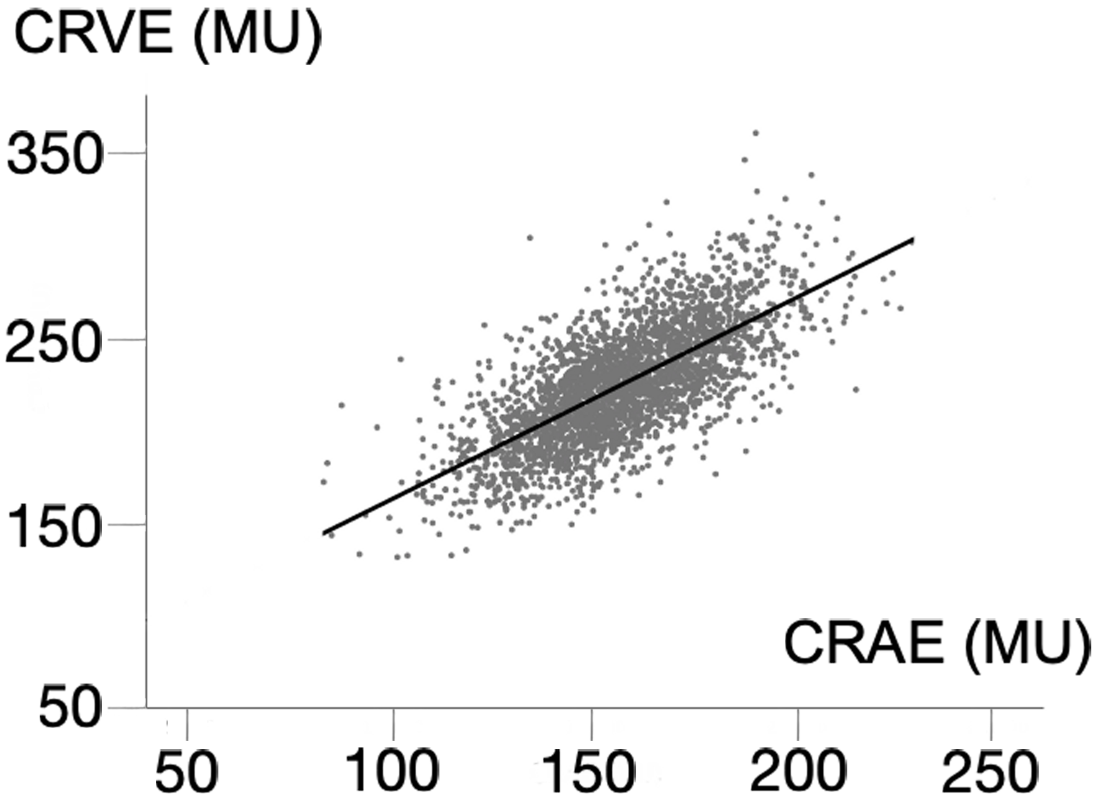

ESM Figure 2. Age- and sex-adjusted association between retinal arteriolar diameters and retinal venular diameters. CRAE, central retinal arteriolar equivalent; CRVE, central retinal venular equivalent; MU, measurement unit;
